# Preparation of Polyethylene and Ethylene/Methacrylic Acid Copolymer Blend Films with Tunable Surface Properties through Manipulating Processing Parameters during Film Blowing

**DOI:** 10.3390/polym11101565

**Published:** 2019-09-26

**Authors:** Sarmad Ali, Youxin Ji, Qianlei Zhang, Haoyuan Zhao, Wei Chen, Daoliang Wang, Lingpu Meng, Liangbin Li

**Affiliations:** 1 National Synchrotron Radiation Laboratory, Anhui Provincial Engineering Laboratory of Advanced Functional Polymer Film, CAS Key Laboratory of Soft Matter Chemistry, University of Science and Technology of China, Hefei 230026, China; sarmad@mail.ustc.edu.cn (S.A.); yxji@zzu.edu.cn (Y.J.); qlzh@mail.ustc.edu.cn (Q.Z.); hyzhao@mail.ustc.edu.cn (H.Z.); dlwang@ustc.edu.cn (D.W.); mlp2014@ustc.edu.cn (L.M.); lbli@ustc.edu.cn (L.L.); 2 School of Materials Science and Engineering, The Key Laboratory of Materials Processing and Mold, Ministry of Education, Henan Key Laboratory of Advanced Nylon Materials and Application, Zhengzhou University, Zhengzhou 450001, China

**Keywords:** PE, EMAA, blown films, haze, wettability

## Abstract

Polymer films based on polyethylene (PE) and ionomer ethylene/methacrylic acid (EMAA) copolymer blend were prepared by film blowing, whose surface properties were tuned by varying processing parameters, i.e., take up ratio (TUR). Blends of PE/EMAA copolymer were firstly prepared by the melt-mixing method, before being further blown to films. The wettability of the film was investigated by measuring the contact angle/water-film encounter time, and optical properties, i.e., the haze and transmittance. The wettability was found to be enhanced with the increase of TUR. So too was the haze, while the transmittance was found to be almost independent of TUR. The XPS and AFM results directly show the increasing polar functional groups (–COO^−^) on the surface and roughness with increasing TUR. Further analysis of the 2D SAXS and WAXS unveiled the origin of the invariant transmittance, which resulted from the minor change of the crystallinity and the monotonic increase of the haze, with TUR resulting from the evolution of crystal orientation. In addition to other post-modification methods, the current study provides an alternative route to prepare large-scale PE films as the template for the advanced potential applications, i.e., covering in the layer of roof, the privacy of protective windows, and multitudes of packaging.

## 1. Introduction 

Polyethylene (PE) film is one of the most widely used polymeric materials, which is attributed to its numerous advantages, i.e., abundant supply, good chemical resistance, low cost, and versatility [1,2,3,4]. However, its lack of reactivity, dyeability, poor wettability, and matting limit its potential applications to some extent. PE films are widely used in multi-product packaging, where optical properties are of paramount importance [5,6,7,8,9,10,11,12]. Films with low haze and high transparency are preferred for packaging and agriculture, since it allows the customers to see inside clearly. However, another type of film with extremely high haze, named matte film, has attracted great interest in the packaging industry in recent years, especially for the commercial enterprise of superior quality stuff [13]. This is due to the fact that the matte surface reduces glare for easy viewing and increases the appeal of the products. Moreover, some of the PE films used in packaging applications are in the form of laminate with other materials, i.e., paper and aluminum, where the adhesion property or the high wettability is the key issue in the applications [14,15] The adhesion property mainly depends on the surface free energy and surface morphology. However, PE films have disadvantages in it owing to its nonpolar nature. Modification of the PE surface is necessary to enhance the interaction between PE and other metal foil layers [11,12,13]. 

Besides packaging, another major usage of PE film is in the field of agriculture as greenhouse covers, which provide a favorable environment to increase plant yield [16]. In general, greenhouse films need to be have as high transmittance as possible for the solar spectrum. Thus, films with low haze and high transparency are favored. However, in peculiar cases, like in the areas where there is perennial sunshine with no cloud, the direct incident of natural light (high transmittance) may harm crops and burn their leaves. Under this condition, films with high haze are required. As a result, the application of PE films as greenhouse covers require tuning the optical properties depending on local conditions. Besides the optical properties, the fogging phenomenon is also crucial in the applications of PE films [17]. Drop-like condensation of water on the film leads to the serious lens effect and falls onto the plants, creating an appropriate environment for the multiplication of disease [2]. The so-called antifogging films with a hydrophilic surface are demanded in order to change the condensation shape from drop-like to a water layer, which does not reflect the light and can flow down gradually [2,18]. However, PE films exhibit limitations for greenhouse coverings because of their hydrophobic behavior [17,19].

Based on the above descriptions, for the major demands, the modification of optical properties, adhesion properties, and wettability of PE films is necessary to adapt to the highly increasing requirements of applications. For the film products, transmittance and haze are the key parameters used to describe the optical properties. The former (transmittance) is defined as the ratio of total transmitted light to the incident light, while the latter (haze) is the percentage of transmitted light that deviates from the directly transmitted beam by more than 2.5° [20]. Both the internal structure and surface roughness can influence the haze, which are referred to as bulk (internal) and surface haze, respectively [5,21]. Bulk scattering is caused by the fluctuation of the refractive index on the scale of the light wavelength, which usually results from the formation of the superstructure [5]. The scattering intensity is roughly proportional to the sixth power of the radius, supposing that spherulitic superstructure is generated [22]. Thus, the haze can be tuned though altering the superstructures, including adding nucleating agents, changing temperature, and flow field. The other general way of influencing the optical properties is to reduce the thickness of the film. The thick film attributes the larger spherulite size, which scatters the light at the large angle and results in the higher haze value [23]. Lots of studies on the quantified relation between superstructure effect and light scattering have been carried out [5,6,10,18,24,25,26] Surface haze arises on account of the existence of surface asperities on the scale of light wavelength (several hundred of nanometers), which induces the occurrence of diffuse reflection and refraction. Studies on the blown film surface morphology show that melt flow behavior and crystallization are two main causes to give rise to the surface roughness [5,6,21]. It has been recognized that the major cause of the high haze is surface haze, which comes from the surface roughness, while the effects of bulk haze are relatively minor, in the case of single-component polymer films [5,27].

Various attempts have been made to improve the adhesion properties of PE, such as flame treatment [28], corona treatment [29], plasma treatment [30], and chemical etching [31], which mainly focus on the post-modification of PE films [32]. However, many of these techniques are subject to certain limitations, such as severe conditions, tedious fabrication, expensive reagents, and special equipment. As to the wettability, hydrophilic additives, such as nonionic surfactants (esters of fatty acids and glycerine or sorbitan) are used to obtain the antifog films [18]. However, the antifog effect decays rapidly during the lifetime of the film, as the surfactants are extracted by the condensed water and usually disappear within two years. New formulations with improved antifog effect quality and duration are needed.

An alternative approach to solving the above concerns is polymer blending, which is a practical way of making new materials with outstanding properties [5,6,15]. The final properties of blends depend on the individual component properties, as well as the composition, morphology, interphase, and processing conditions. In the present work, one kind of ionomer resin with oxygen-containing polar groups, partially neutralized with the Na^+^ ions was added to the PE and fabricate of films by blown film extrusion. Results show that the surface properties heavily depend on the take-up ratio (TUR). With the increase of TUR, the haze increases monotonically, while the transmittance remains about the same at a large value. A transformation from hydrophobic to hydrophilic takes place with increasing TUR. By blending and altering the processing parameters, films with desirable surface properties are obtained, which is expected to provide a new methodology for the manufacturing of high-performance films.

## 2. Experimental Section

**Figure 1:**
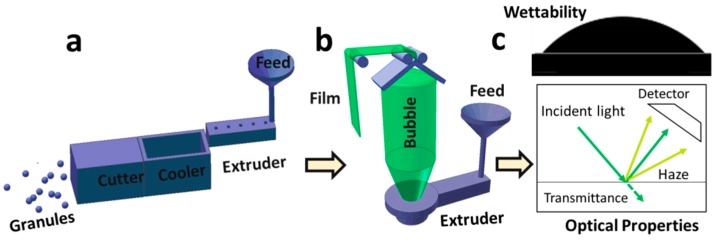
Schematic illustration of the preparation of large-scale polyethylene (PE) and ionomer ethylene/methacrylic acid (EMAA) copolymer films with tunable surface properties: (**a**) preparation of PE/EMAA granules for film blowing; (**b**) film blowing apparatus; (**c**) macroscopic performances, including surface wettability and optical properties.

The whole experimental process is shown in Figure 1.

### 2.1. Materials

**Scheme 1:**
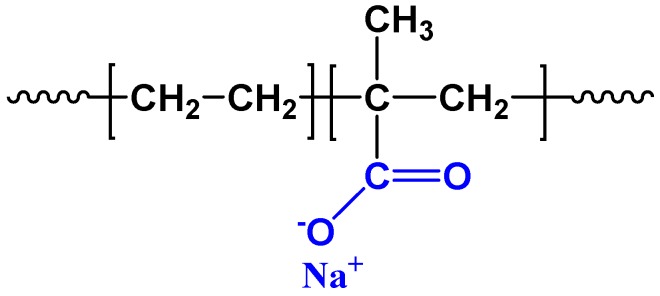
Chemical structure of ethylene/methacrylic acid (EMAA) copolymer (Surlyn).

Polyethylene (PE) used in this study is a mixture of three different kinds of PE: linear low density polyethylenes (LLDPE1, ethylene-octene copolymer, XUS61530, DOW; LLDPE2, ethylene-butene copolymer, 1001AV, ExxonMobil) and low density polyethylene (LDPE, 0725N, TASNEE) with blending ratio of 2:1:1 [33]. The ethylene/methacrylic acid (EMAA) copolymer is ionomer resin (Surlyn 1601, DuPont) with 54% MAA neutralized by the Na^+^ ions. The chemical structure of EMAA copolymer is illustrated in Scheme 1. The basic physical properties of all these polymers are listed in Table 1. PE/EMAA blend was obtained by melt-mixing through corotating twin-screw extruders and cutting into granules. The PE/EMAA (90/10, *wt*/*wt*) sample was chosen in this study.

### 2.2. Film Production

PE/EMAA films with different surface properties were produced by a custom-built film blowing machine. It was equipped with a screw diameter of 25 mm (*L*/*D* = 25) and a die having a diameter of 30 mm with a gap of 1 mm. For the detailed setup of this instrument, refer to our previously reported literature [33,34,35,36]. In this study, a wide range of TUR (from 5 to 30) was adopted, during which, other processing parameters were kept constant with die temperature (220 °C), blow-up ratio (defined as the ratio of the bubble diameter to the die diameter, BUR 2), extrusion speed (2.37 mm/s), and air flux for cooling (as shown in Table 2).

### 2.3. Characterization 

#### 2.3.1. Contact Angle Measurement

Static water contact angle measurement was carried out by Contact Angle Meter SL200B (Solon Tech. Co., Ltd.). A drop of ultra-pure water (2 μL) was placed on the prepared film surface using microsyringe appurtenance and images were captured. The water contact angle was calculated by software CAST 2.0. All measurements were repeated three times.

#### 2.3.2. Haze and Transmittance Measurement

Haze and transmittance were measured with the spherical haze and transmittance meter (SGW-820 Haze-meter, Shanghai INESA Physico-Optical Instrument Co.,Ltd.). All measurements were repeated three times. During the measurements, the thickness of the films was kept same as the actual film obtained after the blown film processing at various TURs. The experimental thickness information, including the theoretical thickness at various TURs, is given at Table S1 and Figure S2.

#### 2.3.3. Atomic Force Microscopy (AFM)

The film surface morphology was characterized by the AFM (Veeco Dimension 3100) with contact mode. The AFM scan size for surface morphology was 2 × 2 μm^2^. All samples were tested directly without further treatment.

#### 2.3.4. SAXS and WAXS Measurements

SAXS measurements were carried out at an in-house small-angle X-ray scattering system with a vertical layout. A micro-focus X-ray source (Genix^3D^ Cu, wavelength *λ* = 1.54 Å) equipped with a PILATUS 300k detector (487 × 407 pixels with a pixel size of 172 × 172 μm^2^) was employed to collect the 2D SAXS data. WAXS measurements were performed by an in-house set up with the help of an image plate detector (Mar 345, 3072 × 3072 pixels with a pixel size of 150 × 150 μm^2^) attached to a micro Cu Kα X-ray source (Incoatec, GmbH, *λ* = 1.54 Å). All SAXS and WAXS patterns were collected within 15 min with a sample-to-detector of 2250 mm and 352 mm, respectively [37,38].

Fit2D software from the European Synchrotron Radiation Facility was used to analyze the data [39,40]. The 2D SAXS and WAXS patterns were integrated to obtain 1D scattering profiles as a function of the scattering vector

(1) q=4π(sin θ)/ λ

where *q* is the module of scattering vector, 2θ the scattering angle, and *λ* is the X-ray wavelength.

The crystallinity *χ*_c_ is calculated as follows:

(2) $$\chi_{c}=\frac{\sum A_{c}}{\sum A_{c}+\sum A_{a}}\times 100\%$$

where *A*_c_ and *A*_a_ are the integrated areas of crystalline and amorphous components, respectively, which are obtained through the multipeak deconvolution of 1D integrated WAXS curve.

The long period *L* was calculated from the *q*_max_ of the 1D SAXS curves:

(3) $$L=\frac{2\pi }{q_{max}}.$$

The orientation parameter of the lamellar stack (*f*) was estimated from the full width at half maximum (FWHM) of the azimuthal-integrated intensity distribution of the 2D SAXS patterns, according to the following equation:

(4) $$f=\frac{180-\mathrm{FWHM}}{180}$$

where *f* equals to 1 when all lamellae are perpendicular to the flow direction, while 0 for no preferred orientation situation.

## 3. Results and Discussion

**Figure 2:**
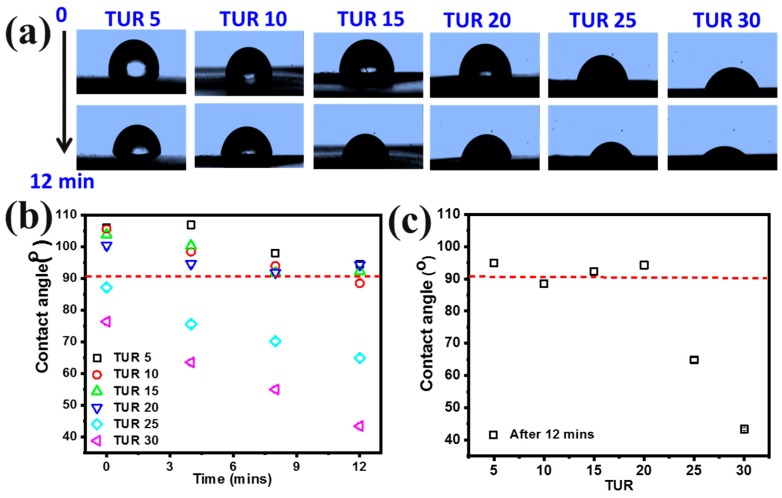
(**a**) Photographs of the water droplet on different PE/EMAA film surfaces; (**b**) contact angle as a function of time at various take up ratios (TURs); (**c**) static contact angle after 12 min.

Blown films wettability: Figure 2a shows the representative photographs of the water droplets on different film surfaces at the time of 0 min and 12 min for the all TURs. It could be observed that the contact area of the droplet increased with the increase of both TUR and time, which reflects increasing of surface wettability. Figure 2b summarizes the quantitative analysis of the time dependence of the contact angle *θ*. The contact angle *θ* showed a similar tendency, i.e., the monotonic reduction in the contact angle *θ* with time and the decreasing rate increasing with the TURs. Within the detection window (<12 min), PE/EMMA films obtained under TUR < 20 had a final contact angle *θ* > 90°, while those obtained under TUR > 20 showed *θ <* 90°. Such a phenomenon should be attributed to the surface modification introduced by varying TURs. Increasing TUR can significantly increase the number of the polar functional groups of EMAA on the film surface, since only EMAA contains hydrophilic carboxylic groups, while PE is strongly hydrophobic. Such a conclusion holds for longer measurement time, as shown in Figure 2c, where the contact angles of different films were obtained after 12 min. In all, the surface wettability of PE/EMAA film from moderate hydrophobic to strongly hydrophilic can be manipulated through varying external processing parameters (TUR). 

**Figure 3:**
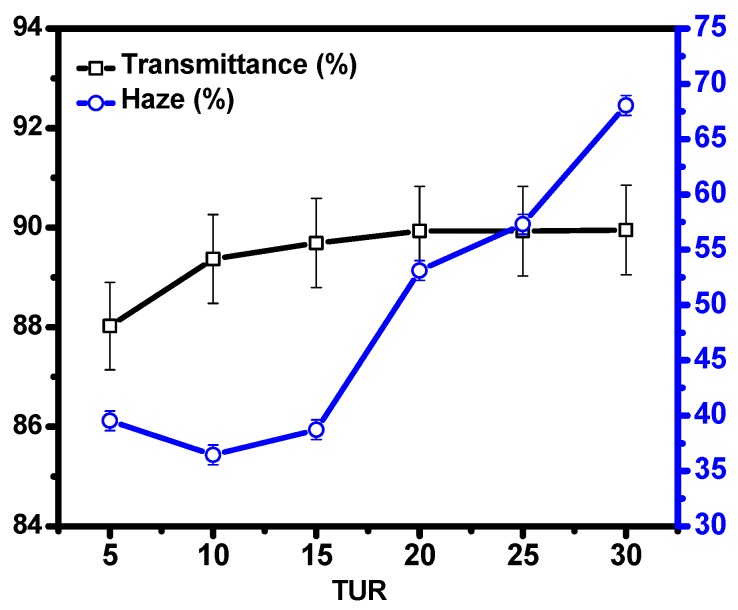
Haze and transmittance of PE/EMAA films at various TURs.

Optical property: The optical property of blown film is crucial in agriculture, where haze and transmittance are two key measurable parameters, as mentioned above [5,6]. The total haze and transmittance of PE/EMAA blown film at various TURs are shown in Figure 3. The transmittance slightly increased from 88% to 89% with the increase of TUR. Such a minor change of transmittance suggests the TUR does not significantly change the transmittance of film. Considering the haze, no apparent change was observed for a film with TUR < 15. It increased dramatically with the TURs from 39.53% (TUR = 15) to 68.05% (TUR = 30). To unveil the origin of the different performances of haze and transmittance as a function of TUR, it is desirable to find key factors determining these two properties. Huck et al. found that the haze actually originates mainly from the surface irregularities of the films, whereas the transmittance is mainly the function of the inner crystallinity [21]. The almost invariable transmittance suggests the inner structure of the PE/EMAA film, i.e., crystallinity, may remain almost unchanged with variable TURs, which will be further elucidated later. For the haze, the dramatic increase was closely related to the surface properties of PE/EMAA, indicating the large variation of surface properties, i.e., roughness, induced by variable TURs. There are two possible mechanisms for the change of haze: one is the extrusion haze and the other is the crystallization haze [5]. The former is related to the polymer melt and just exists in relation to the die, whereas the latter is related to the flow-induced crystallization close to the surface. Since only TUR was changed during the whole preparation process, the crystallization haze should be the dominant mechanism for the change of haze. Increasing TUR could lead to the strengthening of the flow field, and this could cause different anisotropy of crystal. Besides polymer structure, another important concern is related to the film thickness. The film thickness decreases with the increase of the TUR, as shown in Table S1 and Figure S2. The nonmonotonic dependence of film thickness on TUR suggests the complicated nature of PE/EMAA blend film, especially at higher TUR regions (TUR > 20). However, direct experimental evidence tracking the structural evolution during film blowing is necessary to clarify such a phenomenon and its relation with the haze, which will be published elsewhere.

From the film wettability and optical properties, it was concluded that: at the lower TURs (5 to 20), the film surface properties are slightly changed, while with the increase of TUR up to 25 and 30, the rapid decrease in the contact angle and sharp increase in the haze are exhibited by the films. Both of these properties are closely related to the surface properties, i.e., surface roughness and distribution of polar functional groups, and inner film structure, i.e., crystallinity and crystal orientation. The detailed characterization of the surface properties was accomplished based on the XPS and AFM, and that of the inner structure was by WAXS and SAXS.

**Figure 4:**
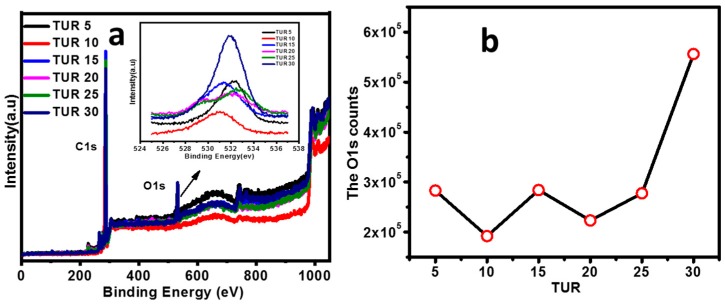
(**a**) Surface composition survey by XPS scans at various TURs; (**b**) calculation of percentage atomic concentration of functional group by O_1s_ peaks.

Chemical composition of blown film surface: X-ray photoelectron spectroscopy (XPS) is one of the most suitable techniques for the film surface composition/enrichment analysis, which is quantified in terms of peak position and peak intensity. The peak position contains characteristic features of different atoms in different states, and corresponding peak intensities are proportional to their quantities. As a result, XPS can quantify the elemental composition of the film surface. Figure 4a presents the XPS whole survey results, including the enlarged O_1s_ lineshape of PE/EMAA films obtained at different TURs. Two sharp peaks were observed at the binding energies of 289–290 eV and 531.5–533 eV, which were assigned to C_1s_ lattice and O_1s_, respectively [41]. No apparent peak shift was observed for C1s (289–290 eV, Figure S3). For O_1s_ (531.5–533 ev), increasing TUR led to the transition from singlet to doublet peak, whereas further increasing TUR resulted in the emergence of the doublet peak. As mentioned above, only EMAA contains the polar functional group—carboxylic group. The binding energy of O_1s_ for a single C–O bond falls in the binding energy of 531.5–532 eV, whereas that of the double carbonyl organic bond C=O lies in the range of 532–533 eV. [42]. Therefore, such doublet peak could be attributed to the coexistence of single and double bonds on the film surface. The intensity of O_1s_ is proportional to the concentration of EMAA on the surface. The counts of functional carboxylic groups were calculated from the area under the peak O_1s_. Figure 4b shows the atomic concentration of O_1s_ contents. It shows that the O_1s_ count remained almost constant within low TURs (5 to 20), but increased dramatically for film obtained at TUR = 30. This suggests more functional polar groups (–COO^−^) appear on the surface at the high flow condition.

**Figure 5:**
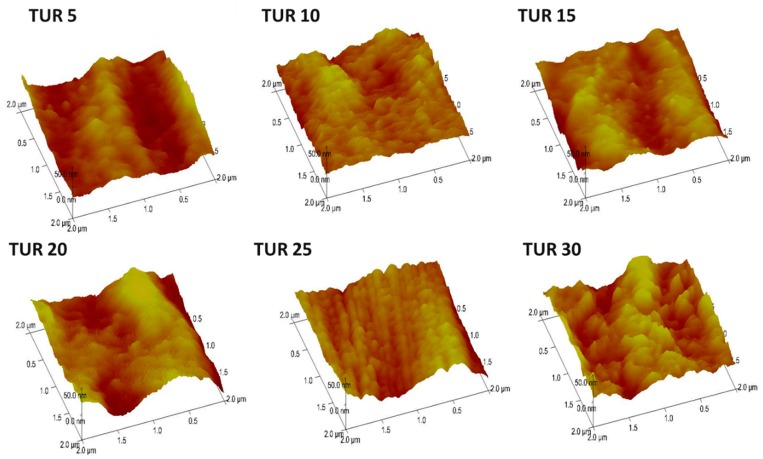
AFM height images of PE/EMAA films obtained at various TURs.

**Figure 6:**
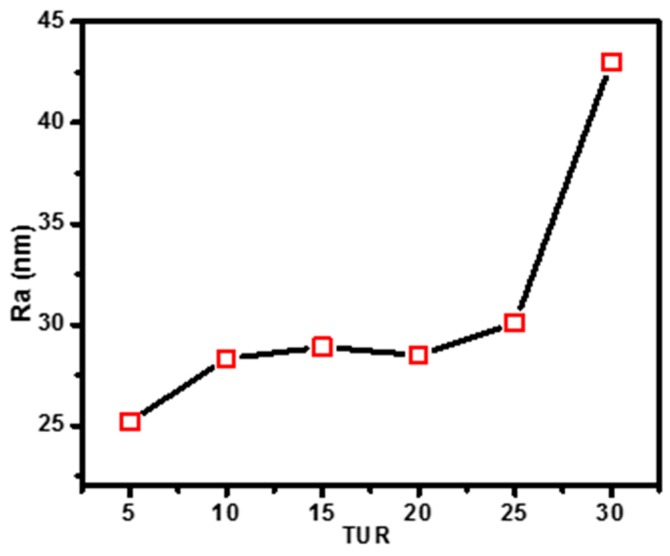
The surface roughness *R*_a_ of different PE/EMAA films as characterized by AFM.

Surface roughness of blown film: The atomic force microscopy (AFM) was used to analyze surface film texture and roughness of the PE/EMAA blown films. Figure 5 shows the topographic images of PE/EMAA films obtained under different TURs. Generally, all AFM height images showed irregular surface morphology, and with increasing TUR, more irregular stacked domains appeared above the surface. The orientation difference of the lamellae on the surface obtained under different TURs could also be observed in the AFM phase images (Figure S1). The quantitative analysis of the AFM images resulted in the surface roughness (*R*_a_), as shown in Figure 6. The roughness increased monotonically with the TUR, and changed abruptly at TUR = 30 (*R*_a_ = 43 nm). The almost double increase in surface roughness indicates the significant modification of surface induced by TUR. The film texture, i.e., crystal morphology and lamellar orientation, is not clearly characterized by AFM, which is later characterized by X-ray scattering.

Above crystal surface characterization results have shown that increasing TUR can increase the quantity of polar functional groups and surface roughness. The decreasing contact angle was well consistent with the increase of carboxylic groups on the surface. With respect to the optical properties, the haze was initially thought to result from the coupling influence of surface composition and texture morphology. Since the haze increases continuously with the increase of TUR, whereas both the concentration of carboxylic groups and surface roughness remains almost constant with TUR < 25, it suggests that the haze of final polymer films are not solely determined by the surface composition and surface roughness. Another possible origin is the orientation of the crystal lamellae, as discussed below.

**Figure 7:**
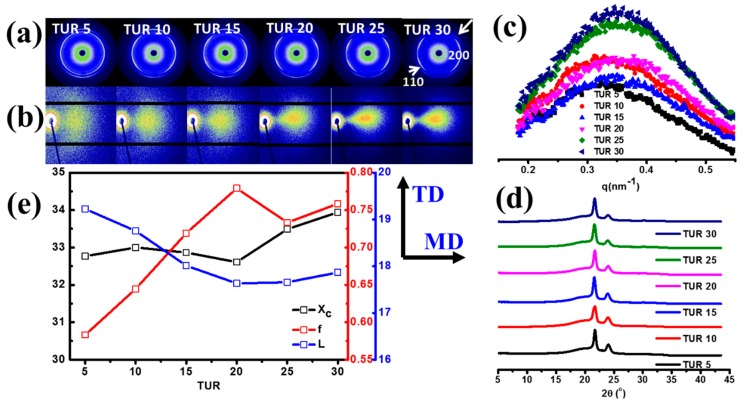
**(a)** Ex situ 2D WAXS diffraction images; (**b**) 2D-SAXS scattering patterns, the machine direction (MD) is horizontal; (**c**) the 1D integrated SAXS curves; (**d**) 1D integrated WAXS curves; (**e**) the crystallinity *χ*_c_ (%), orientation factor *f,* and long period *L* (nm) calculated from the WAXS and SAXS 1D curves, respectively, at various TURs.

Internal hierarchical structure: The internal structure of the PE/EMAA films at various TURs were characterized by ex situ WAXS and SAXS, as shown in Figure 7. The PE/EMAA films exhibited two sharp peaks at 2*θ* = 21.6° and 24.08°, corresponding to (110) and (200) crystal planes of polyethylene in orthorhombic structure, respectively (Figure 7a). The WAXS scattering ring first appeared for film obtained at TUR = 5, which indicates the randomly distributed lamellae. Further increase of TUR led to the four-spot patterns along the machine direction (MD). This suggests highly oriented crystal is formed under the strong flow field [43,44,45]. Such transition from low anisotropy to high orientation was further confirmed by the 2D SAXS patterns, as shown in Figure 7b. Quantitative analysis of the WAXS and SAXS patterns was accomplished based on the 1D integrated curves, as shown in Figure 7c,d. The lamellar thicknesses *L* and crystal orientations *f* of different films can be calculated based on the *q*_max_ and anisotropy calculation in 1D SAXS curves (eq. 2–3), while the crystallinity *χ*_c_ can be obtained through the multi-peak deconvolution of 1D WAXS curves (eq. 1). As summarized in Figure 7e, the crystallinity (*χ*_c_) increased negligibly from 32.8% to 33.9%, so too did the long period *L* slightly decrease from 19 to 18 nm. However, the crystal orientation (*f*) increased monotonically with increasing TUR from 0.58 to 0.75. Such moderate increase of the crystallinity should be attributed to the coupling influence of both external flow field and the temperature gradient. Although strong flow field favors the formation of oriented crystals, the faster cooling rate will also happen, which slows down the crystallization rate. Considering the minor change of the transmittance and the significant increase of the haze with increasing TUR, it can be concluded that the transmittance is mainly determined by the crystallinity and crystal orientation, whereas the haze is significantly influenced by crystal orientation. 

## 4. Conclusions

In this article, the PE/EMAA films with tunable surface properties were prepared through manipulating the processing parameter (TUR) during film blowing. The film surface exhibited strong hydrophilic behavior (contact angle = ca. 45°) and higher optical haze (60%) at the higher taking up ratios. The inner structure of film influenced slightly on the total haze (supporting previous studies). The super-spherulitic structure near to the film surface was the major contributor to the large scattering from the film surface roughness, which further increased total haze at higher TURs. The roughness was caused by the EMAA enrichment on the surface, which helped to increase the film hydrophilicity effectively. The current study provides an alternative method to one-step prepare large-scale PE film with polar functional groups and can be used as a template for advanced film capable of application in various fields, i.e., agriculture and private stuff packaging.

## Figures and Tables

**Table 1:** Typical molecular characteristics and rheological properties of polymers.

Formula	Density (g/cm^3^)	Melt Flow Index (g/10 min)	*M*_w_ (kg/mol)	PDI
*LLDPE1*	0.917	0.8	133	4.2
LLDPE2	0.919	1.0	151	4.9
LDPE	0.923	0.75	109	5.1
EMAA	0.940	1.3	—	—

**Table 2:** The processing parameters of blown films (*v*_t_ is the taking up speed).

	TUR-5	TUR-10	TUR-15	TUR-20	TUR-25	TUR-30
v_t_ (mm/s)	11.9	23.9	35.4	47.3	59.3	71.1
TUR	5	10	15	20	25	30

## Supplementary Materials

The following are available online at https://www.mdpi.com/2073-4360/11/10/1565/s1, Figure S1: AFM phase images of PE-EMAA film obtained at different TURs, Figure S2: Film thickness vs. the inverse of TUR, Figure S3 XPS C1s lineshape at various TURs. Table S1. Film Thickness of PE-EMAA films obtained under different TURs.
