# Supplementary material for: Preparation of Polyethylene and Ethylene/Methacrylic Acid Copolymer Blend Films with Tunable Surface Properties through Manipulating Processing Parameters during Film Blowing
Source: Polymers (Basel). 2019 Sep 26;11(10):1565. doi: 10.3390/polym11101565 (PMC6836035; doi:10.3390/polym11101565)
Supplement: Supplementary file 1 [file polymers-11-01565-s001.pdf]

polymers

MDPI

Supporting Information

Article

Preparation of Polyethylene and Ethylene/Methacrylic Acid Copolymer Blend Films with Tunable Surface Properties through Manipulating Processing Parameters during Film Blowing

Sarmad Ali [1,†], Youxin Ji [1,2,†] Qianlei Zhang [1], Haoyuan Zhao [1], Wei Chen [1,*], Daoliang Wang [1], Lingpu Meng [1] and Liangbin Li [1]

[1] National Synchrotron Radiation Laboratory, Anhui Provincial Engineering Laboratory of Advanced Functional Polymer Film, CAS Key Laboratory of Soft Matter Chemistry, University of Science and Technology of China, Hefei 230026, China

[2] School of Materials Science and Engineering, The Key Laboratory of Materials Processing and Mold, Ministry of Education, Henan Key Laboratory of Advanced Nylon Materials and Application, Zhengzhou University, Zhengzhou 450001, China

* Correspondence: wc003@ustc.edu.cn

† These authors contributed equally to this work.

Received: 10 September 2019; Accepted: 24 September 2019; Published: date

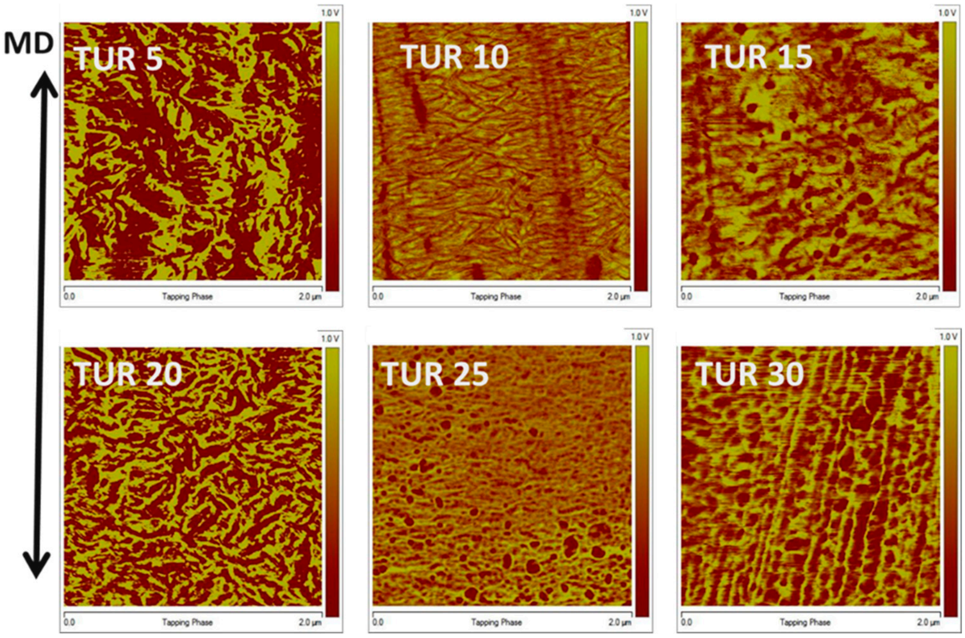

Figure S1. AFM phase images of PE-EMAA film obtained at different TURs.

Table S1. Film Thickness of PE-EMAA films obtained under different TURs.

TUR	Experimental Thickness* (μm)
5	112.0
10	82.0
15	65.0
20	44.0
25	34.0
30	28.0

*Error ±10%.

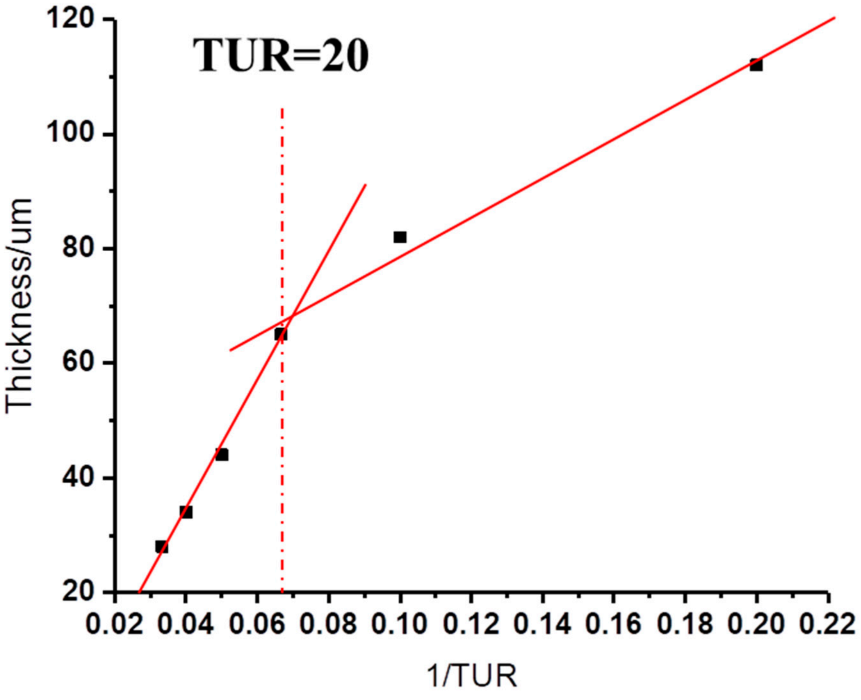

Figure S2. Film thickness vs. the inverse of TUR.

The film thickness is inversely proportional to TUR, and can be expressed as[1]

$$\mathrm{H} = \frac{(a_o^2 - a_i^2)}{2a_0}\left(\frac{\rho_m}{\rho_s}\right)\frac{1}{(BUR)(TUR)} \quad (1)$$

where a_o and a_i are the outer and inner radiuses of die, ρ_m and ρ_s are the densities of film in melt and solid states. However, the measured thicknesses are not monotonically proportional to 1/TUR. This suggests the complicated nature of the PE/EMAA blends, where other parameters need to be considered. The origin of such phenomenon requires detailed microstructure evolution information of the blend films, which will be published elsewhere.

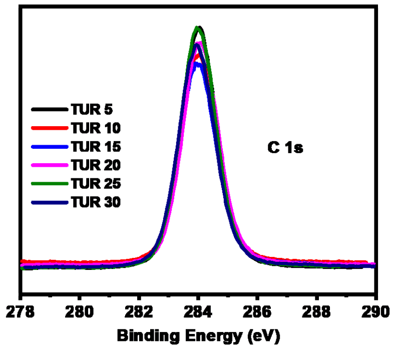

Figure S3. XPS C_{1s} lineshape at various TURs.

References:

1. Kwack, T. H.; Han, C. D. Rheology-processing-property Relationships in Tubular Blown Film Extrusion. II. Low-pressure Low-density Polyethylene. *J. Appl. Polym. Sci.* **1983**, *28* (11), 3419–3433.
